# Amyloid Precursor Protein Binding Protein-1 Modulates Cell Cycle Progression in Fetal Neural Stem Cells

**DOI:** 10.1371/journal.pone.0014203

**Published:** 2010-12-02

**Authors:** Yuyoung Joo, Sungji Ha, Bo-Hyun Hong, Jeong a Kim, Keun-A Chang, Hyunjeong Liew, Seonghan Kim, Woong Sun, Joung-Hun Kim, Young Hae Chong, Yoo-Hun Suh, Hye-Sun Kim

**Affiliations:** 1 Department of Pharmacology, College of Medicine, Seoul National University, Seoul, Republic of Korea; 2 Department of Anatomy, School of Medicine, Korea University, Seoul, Republic of Korea; 3 Department of Life Science, Pohang University of Science and Technology, Pohang, Republic of Korea; 4 Department of Microbiology, School of Medicine, Ewha Womans University, Seoul, Republic of Korea; 5 Seoul National University Bundang Hospital, Seoul National University College of Medicine, Sungnam, Republic of Korea; University of Victoria, Canada

## Abstract

Amyloid precursor protein binding protein-1 (APP-BP1) binds to the carboxyl terminus of the amyloid precursor protein (APP) and serves as the bipartite activation enzyme for the ubiquitin-like protein, NEDD8. In the present study, we explored the physiological role of APP-BP1 in the cell cycle progression of fetal neural stem cells. Our results show that cell cycle progression of the cells is arrested at the G1 phase by depletion of APP-BP1, which results in a marked decrease in the proliferation of the cells. This action of APP-BP1 is antagonistically regulated by the interaction with APP. Consistent with the evidence that APP-BP1 function is critical for cell cycle progression, the amount of APP-BP1 varies depending upon cell cycle phase, with culminating expression at S-phase. Furthermore, our FRET experiment revealed that phosphorylation of APP at threonine 668, known to occur during the G2/M phase, is required for the interaction between APP and APP-BP1. We also found a moderate ubiquitous level of APP-BP1 mRNA in developing embryonic and early postnatal brains; however, APP-BP1 expression is reduced by P12, and only low levels of APP-BP1 were found in the adult brain. In the cerebral cortex of E16 rats, substantial expression of both APP-BP1 and APP mRNAs was observed in the ventricular zone. Collectively, these results indicate that APP-BP1 plays an important role in the cell cycle progression of fetal neural stem cells, through the interaction with APP, which is fostered by phopshorylation of threonine 668.

## Introduction

Amyloid precursor protein binding protein-1 (APP-BP1) has been known to interact with the intracellular carboxyl (C-) terminus of the amyloid precursor protein (APP), the precursor protein of amyloid beta peptide (Aβ), which is the main component of neuritic plaques in Alzheimer's disease (AD) [1],[2],[3].

APP-BP1, like APP, is ubiquitously expressed in neural and non-neural tissues [4]. The intracellular C-terminal domain of APP interacts with several proteins, including the Fe65 protein family [5], JNK interacting protein 1 [6], X11 [7], APP-BP1 [4], and others. Although extensive research has been done to characterize the normal physiological function of APP and its interaction with the proteins described above, there are many aspects that still require clarification.

APP-BP1 is localized to human chromosome 16 band q22 [4] and acts as one component of the bipartite activating enzyme for the ubiquitin-like small molecule, NEDD 8 [8],[9],[10]. Upon binding to Uba3, which is homologous to the carboxyl terminus of E1, APP-BP1 acts as an activating enzyme, thus activating NEDD8. APP-BP1/Uba3 also interacts with the N-terminus of the conjugating enzyme Ubc12, which is analogous to E2 in the uniquitination pathway [9], [10], [11], [12].

Neddylation is involved in various cellular functions including cell cycle progression [13], [14], [15]. Several targets for neddylation exists in mammalian cells, including the cullin (Cul) family members, a major constituent of the ubiquitin-ligase, Skp-1-Cul-1-F box (SCF) complex [16], [17]. SCF ubiquitin ligase targets p27, the cyclin-dependent kinase (cdk) inhibitor, for degradation during the transition of cells from the G_0_/G_1_ phase to the S phase of the cell cycle [18], and also regulate PDCD4, Cdc25A, Claspin, Wee1, Emi1, cyclin E, and cyclin D1, all of which are key substrates within the cell division cycle [19], [20].

Overexpression of APP-BP1 in primary neurons induces apoptosis and increases DNA synthesis [21]. In addition, up-regulated APP-BP1 expression has been observed in the lipid rafts in the hippocampi of AD brains, when compared with age-matched control brains [22].

In this study, we focused on the role for APP-BP1 in neural stem cell cycle progression, and demonstrated that APP-BP1 is critically required for cell cycle progression. This action of APP-BP1 is antagonistically regulated by the interaction with APP. Additionally, phosphorylation of APP at the threonine 668 residue was found to be required for the interaction with APP-BP1.

## Results

### APP-BP1 knockdown induced cell cycle arrest at G1 phase

To investigate whether APP-BP1 affects the cell cycle progression of fetal neural stem cells, we first tested the knockdown effect of siRNAs for APP-BP1 (APP-BP1 siRNA) by comparing them with non-targeting control siRNA. We found that treatment with the targeted siRNA for 72 h reduced the protein level of APP-BP1 by about 50% (Figure S1A). An analysis of the cell cycle was then performed using 10,000 cells pulled from three treatment groups: untreated cells, non-targeting control siRNA-, or APP-BP1 siRNA-transfected fetal neural stem cells (passage 8) and HEK 293 cells, employing a FACS. After the cells were treated with either the vehicle, non-targeting siRNA or APP-BP1 siRNA for 72 h, cells were harvested and fixed with 70% ethanol for 1 h, and stained with PI for 1 h. FACS was performed as described in Materials and Methods.

**Figure 1:**
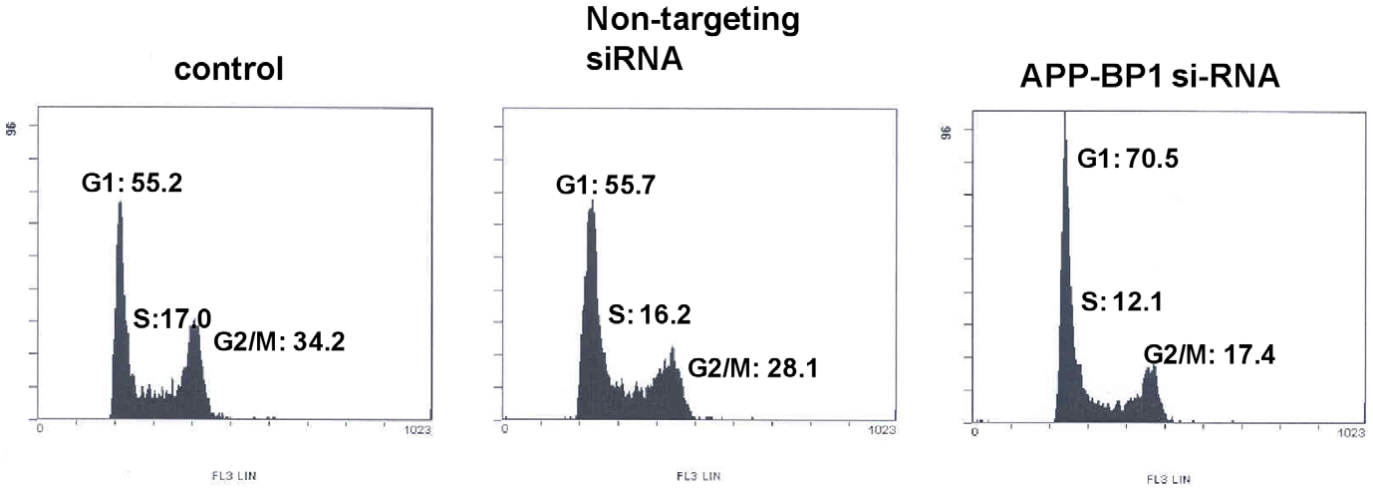
APP-BP1 knockdown arrests cell cycle at G1-phase in fetal neural stem cells. The DNA content of the siRNAs treated fetal neural stem cells (Passage 8) was evaluated by flow cytometry with a FACScan instrument. Each group represents the status of the cell cycle for 10,000 cells.

We found that the cell population at G1 phase had increased in both HEK 293 cells (data not shown) and fetal neural stem cells (Fig. 1). The cell population at G1 phase in untreated and non-targeting siRNA-treated fetal neural stem cells was 48.8 and 55.7%, respectively. In APP-BP1 siRNA-treated cells, the cell population at G1 phase was 70.5% in fetal neural stem cells. The percentage of the cell population at S phase was 17.0%, 16.2% and 12.1% in untreated, non-targeting siRNA-treated and APP-BP1 siRNA-treated cells, respectively. The percentage of the cell population at the G2/M phase was 34.2%, 28.0% and 17. 4% in untreated, non-targeting siRNA-treated and APP-BP1 siRNA-treated cells, respectively (Fig. 1). These results demonstrated that APP-BP1 is required for the cell cycle progression from G1 to S phase.

### APP-BP1 is required for the proliferation in fetal neural stem cells

**Figure 2:**
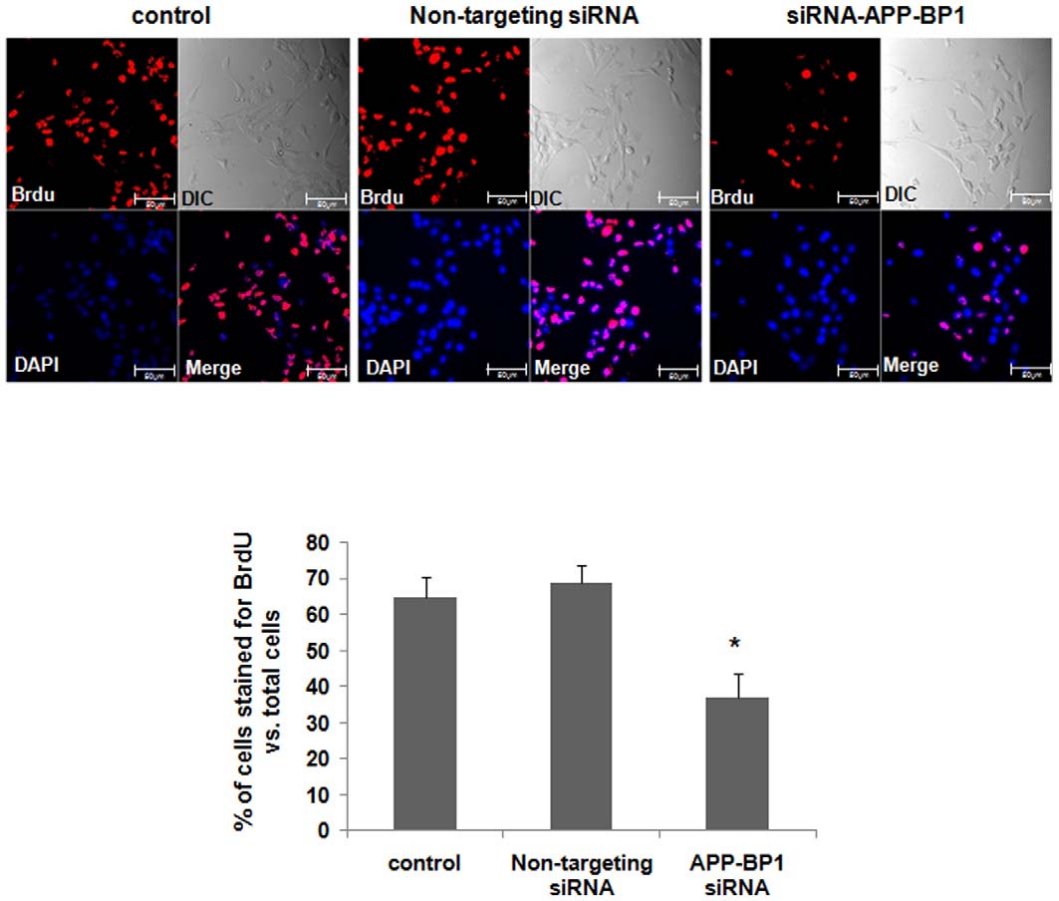
siRNA-mediated APP-BP1 knock-down significantly decreases BrdU-incorporated fetal neural stem cells. Fetal neural stem cells were subcultured in 6-well plates, in proliferating media, and then treated the cells with 10 nM siRNAs. After 24 h, the cells were treated with BrdU (1 µM) for 24 h. We then dissociated the neurospheres, and transferred the cells to new 6-well plates with 12 mm glass coverslips pre-coated with laminin (20 µg/ml). The cells were cultured in the differentiation media without growth factors for 30 min and assessed with immunocytochemistry using an anti BrdU and Cy3-secondary antibodies. DAPI (1 µM) staining shows the location of the nucleus (blue). DIC represents differential image contrast. BrdU immunoreactive cells were calculated in five independent experiments (10 fields were evaluated per each experiment) with respect to the ratio of BrdU stained cells to total cells. Data are expressed as mean ± SEM values. One-way ANOVA was used to determine statistical significance at *p*<0.05 by post hoc analysis via Duncan's test. Scale bars = 50 µm.

We next determined whether APP-BP1 knockdown affected the proliferation of fetal neural stem cells. Bromodeoxyuridine (5-bromo-2-deoxyuridine, BrdU) was used to examine proliferation. BrdU can be incorporated into the newly synthesized DNA of replicating cells during the S phase of the cell cycle, where it substitutes for thymidine during DNA replication. The cells were treated with 30 µM BrdU after vehicle or siRNAs treatment for 24 h. After 24 h, cells were dissociated and incubated for 10 min before being placed on laminin coated coverslips and examined using immunocytochemistry with an anti-BrdU antibody. We calculated the ratio of BrdU positive cells to the total number of cells in 5 independent experiments. A total of about 700–1100 cells were analyzed from each experiment. We found that this ratio was significantly decreased in APP-BP1 siRNA-transfected fetal neural stem cells (36.92±6.75%), compared to untreated cells (64.74±5.95%) (Fig. 2).

### APP knockdown increased proliferation in fetal neural stem cells

**Figure 3:**
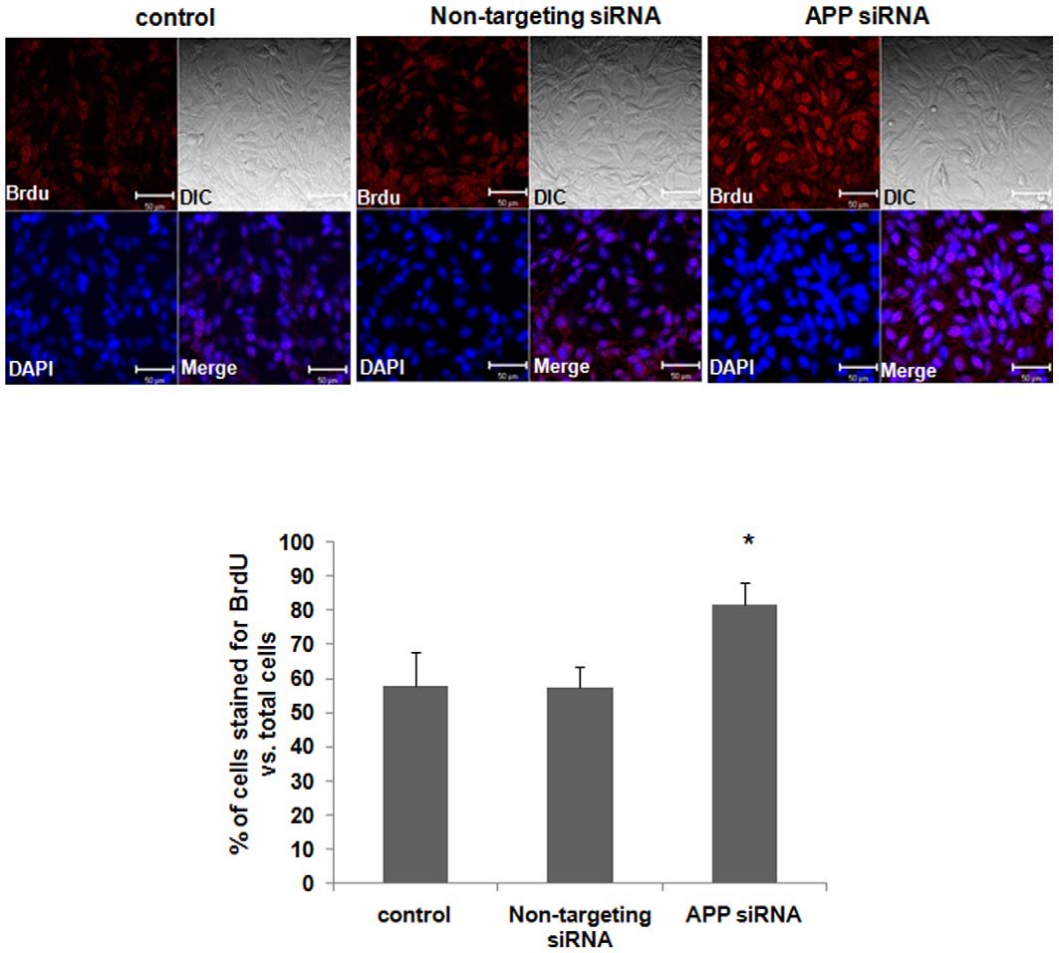
siRNA-mediated APP knock-down significantly increases the proliferation of fetal neural stem cells. Fetal neural stem cells were subcultured in 6-well plates, in proliferating media, and then treated the cells with 10 nM siRNAs. After 24 h, the cells were treated with BrdU (1 µM) for 24 h. We then dissociated the neurospheres, and transferred the cells to new 6-well plates with 12 mm glass coverslips pre-coated with laminin (20 µg/ml). The cells were cultured in the differentiation media without growth factors for 30 min and assessed with immunocytochemistry using an anti BrdU and Cy3-secondary antibodies. DAPI (1 µM) staining shows the location of the nucleus (blue). DIC represents differential image contrast. BrdU immunoreactive cells were calculated in five independent experiments (10 fields were evaluated per each experiment) with respect to the ratio of BrdU stained cells to total cells. Data are expressed as mean ± SEM values. One-way ANOVA was used to determine statistical significance at *p*<0.05 by post hoc analysis via Duncan's test. Scale bars = 50 µm.

**Figure 4:**
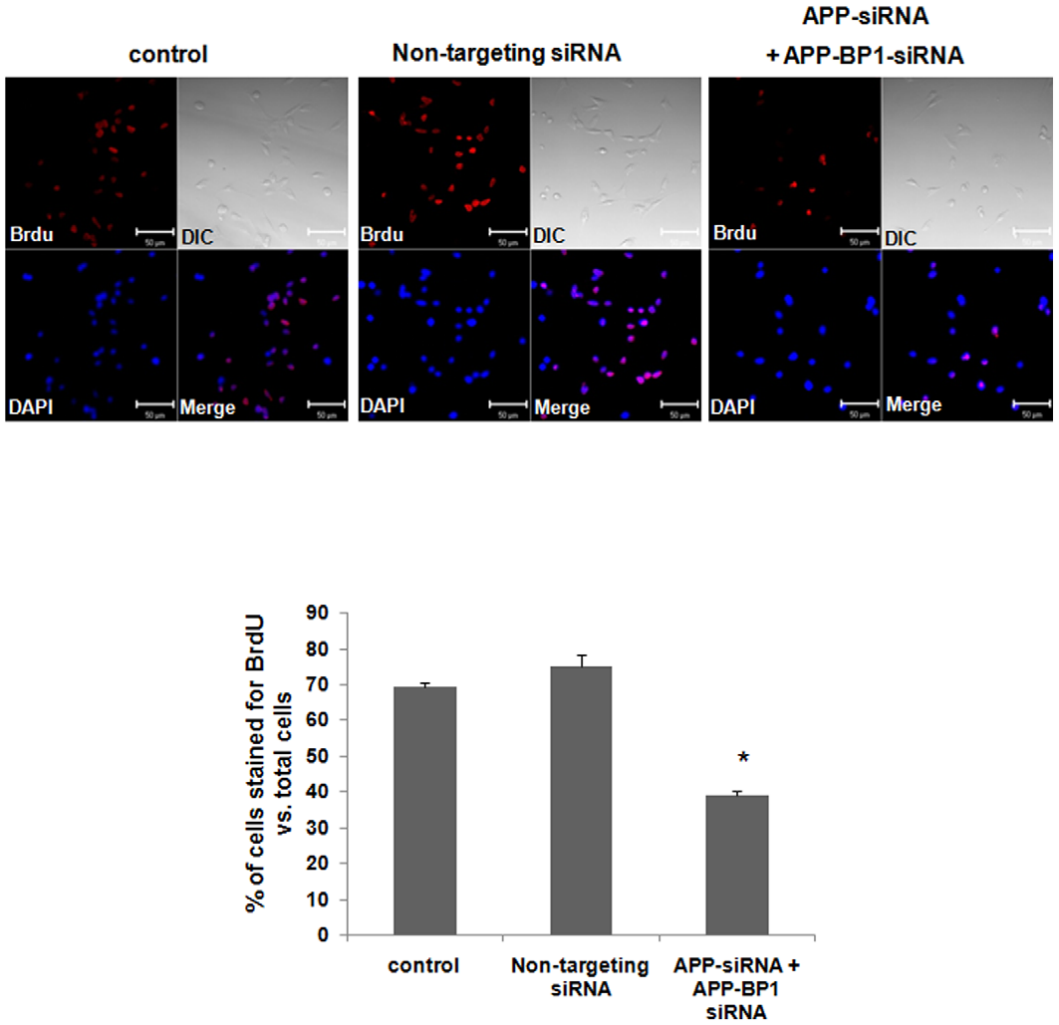
siRNA-mediated knock-down of APP-BP1 and APP significantly down-regulates the proliferation of fetal neural stem cells. Fetal neural stem cells were subcultured in 6-well plates, in proliferating media, and then treated the cells with 10 nM siRNAs. After 24 h, the cells were treated with BrdU (1 µM) for 24 h. We then dissociated the neurospheres, and transferred the cells to new 6-well plates with 12 mm glass coverslips pre-coated with laminin (20 µg/ml). The cells were cultured in the differentiation media without growth factors for 30 min and assessed with immunocytochemistry using an anti BrdU and Cy3-secondary antibodies. DAPI (1 µM) staining shows the location of the nucleus (blue). DIC represents differential image contrast. BrdU immunoreactive cells were calculated in five independent experiments (10 fields were evaluated per each experiment) with respect to the ratio of BrdU stained cells to total cells. Data are expressed as mean ± SEM values. One-way ANOVA was used to determine statistical significance at *p*<0.05 by post hoc analysis via Duncan's test. Scale bars = 50 µm.

We also examined whether the knockdown of APP, the binding partner of APP affected the proliferation of fetal neural stem cells using BrdU staining as described above. A total of about 700–1100 cells were analyzed from each experiment. APP siRNA treatment was shown to reduce APP protein level by about 50% (Figure S1B). The ratio of BrdU positive cells to the total number of cells was significantly increased in APP siRNA transfected fetal neural stem cells (81.74±6.54%), compared to untreated control cells (57.97±9.70%) (Fig. 3). We also found that the treatments with both APP and APP-BP1 siRNAs into the cells significantly reduced the BrdU positive cells (39.06±1.39%), compared to untreated control cells (69.55±1.12%) (Fig. 4). These results suggest that APP antagonistically regulates APP-BP1's function in cell cycle progression.

### APP-BP1 expression varied according to cell cycle phase

**Figure 5:**
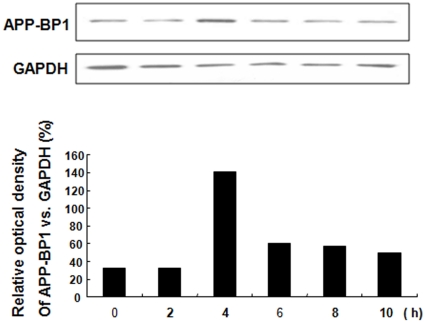
APP-BP1 expression varied according to cell cycle phase. Fetal neural stem cells were synchronized to G1 phase by treatment with 2 mM thymidine for 16 h, and then released from cell cycle arrest by replacing the thymidine-containing media. Cells were harvested at 0, 2, 4, 6, 8, 10 and 12 h, and APP-BP1 protein levels were examined by Western blotting. The blot is representative of three independent experiments. The protein level of GAPDH was examined as a loading control.

Next, we investigated whether APP-BP1 expression was altered according to the cell cycle progression. Fetal neural stem cells were synchronized to the G1 phase by treatment with 2 mM thymidine for 16 h, and then released from cell cycle arrest by replacing the thymidine-containing media. Cells were harvested at 0, 2, 4, 6, 8, 10 and 12 h after cell cycle arrest through treatment with 2 mM thymidine, and the APP-BP1 protein level was examined via Western blotting (Fig. 5). APP-BP1 was increased at 4 h, corresponding to the S phase in FACS measurements (data not shown). Although further experiments needed, this result suggests that APP-BP1 was induced in the S phase and thus, may play a role in S phase or the progression from G1 to S phase.

### The expression of APP and APP-BP1 during developmental stages was investigated

Although the expression profile of APP-BP1 during development in *Drosophila* was reported previously [23], there has been no report on the expression profile of APP–BP1 in mammals. We investigated the mammalian expression pattern of APP-BP1 and APP, based on our hypothesis that APP-BP1 plays a role in accordance with APP.

**Figure 6:**
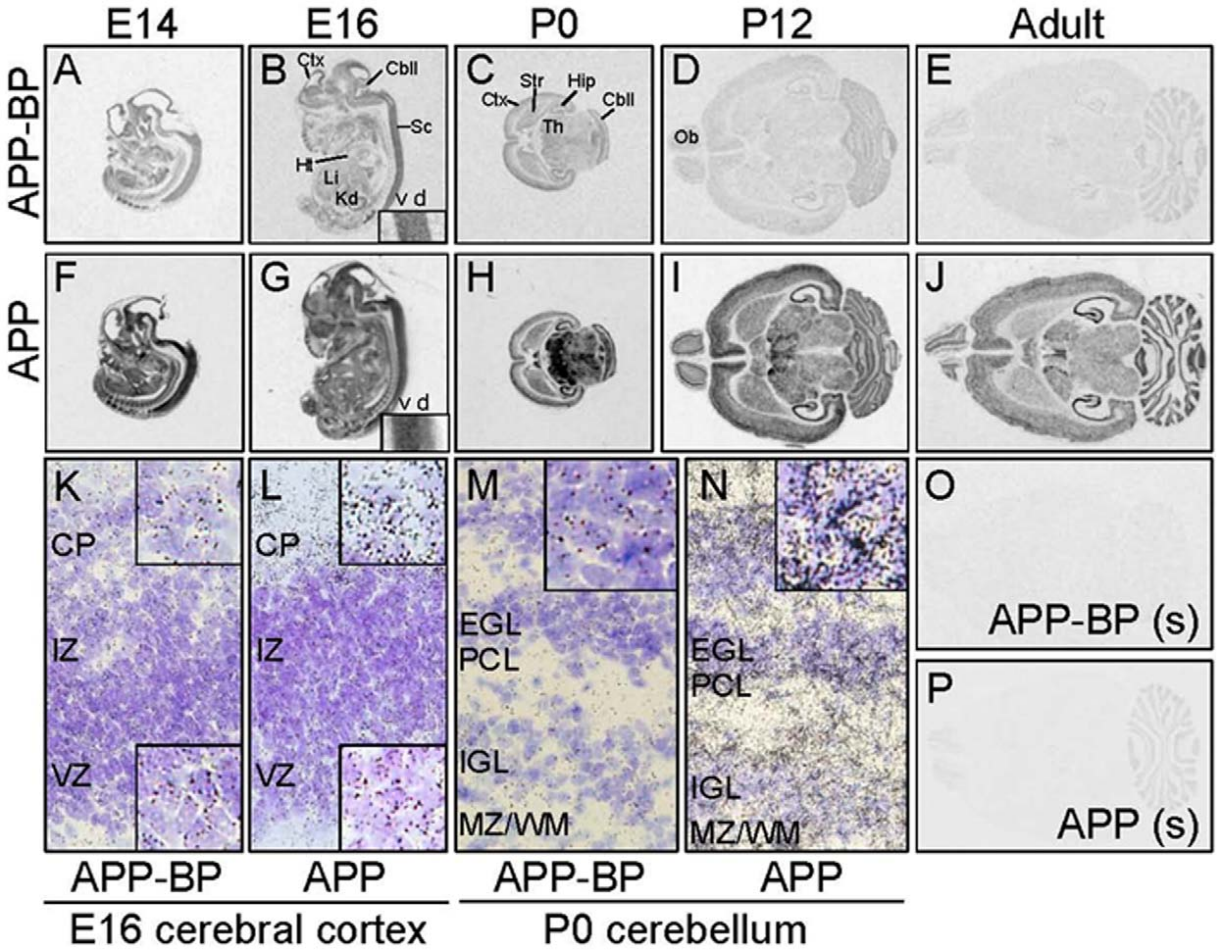
Developmental changes in the expression of APP and APP-BP1 were examined. The distribution of APP (**A–E**) and APP-BP (**F–J**) mRNA was detected by ISH at E14 (**A, F**), E16 (**B, G**) embryos and P0 (**C, H**), P12 (**D, I**), and in adult (**E, J**) brains. Insets in **B** and **G** show a large magnification view of spinal cord. (**K–N**): Emersion-developed black silver grains of APP-BP (**K, M**) and APP (**L, N**) mRNA signal showed distribution in the E16 cerebral cortex (**K, L**) and P0 cerebellum (**M, N**). Insets in **K** and **L** show large magnification view of CP (upper insets) and VZ (lower insets), and the insets in **M** and **N** shows enlarged views of EGL. (**O, P**): Adult brain sections were hybridized with sense probes for APP-BP (**O**) or APP (**P**). Experiments were duplicated, and typical images are shown. Abbreviations: Ctx, cerebral cortex; Cbll, cerebellum; Sc, spinal cord; Ht, heart; Li, liver; Kd, kidney; Str, striatum; Th, thalamus; Hip, hippocampus; Ob, olfactory bulb; v, ventral spinal cord; d, dorsal spinal cord; CP, cortical plate; IZ, intermediate zone; VZ, ventricular zone; EGL, external germinal layer; PCL, Purkinje cell layer; IGL, inner granule cell layer; MZ/WM, marginal zone/white matter.

A moderate level of APP-BP1 mRNA signal was ubiquitously observed in the developing embryonic and early postnatal brains (Fig. 6A–D). However, APP-BP1 expression appeared to be reduced by P12 (Fig. 6D), and only a marginal signal was found in the adult brains (Fig. 6E). Alternatively, strong APP mRNA signals were observed in both the developing and adult brains (Fig. 6F–J). The expression of APP was strong in the ventral region of the spinal cord in the E16 embryo (Fig. 6G, inset). Compared with APP, APP-BP1 expression was ubiquitously observed in the E16 spinal cord (Fig. 6B, inset). In the cerebral cortex of E16 rats, substantial signals for both APP-BP1 (Fig. 6K) and APP (Fig. 6L) mRNAs were observed in the ventiricular zone (VZ) where proliferating neuroepithelial cells are enriched. The expression of APP was stronger in the cortical plate (CP) where post-mitotic neurons reside, whereas the expression level of APP-BP1 in the VZ and CP was similar. On P0, strong expression of APP was observed in the thalamus, hippocampus, and cerebellum. In the P0 cerebellum, the APP signal was especially strong in the external germinal layer (EGL; Figure 4N, inset) and inner granule cell layer (IGL, Fig. 6N), whereas APP-BP1 expression was only marginal. As opposed to APP-BP1 (Fig. 6E), strong expression of APP was maintained in the adult brain (Fig. 6J). In particular, APP expression was strong in the adult hippocampus and cerebellum. These ISH signals appeared to be specific because sense probe hybridization exhibited a markedly reduced or complete lack of signals compared to the antisense hybridizations (Fig. 6O, P).

### Molecular interaction between APP-BP1 and APP was examined by FRET analysis

**Figure 7:**
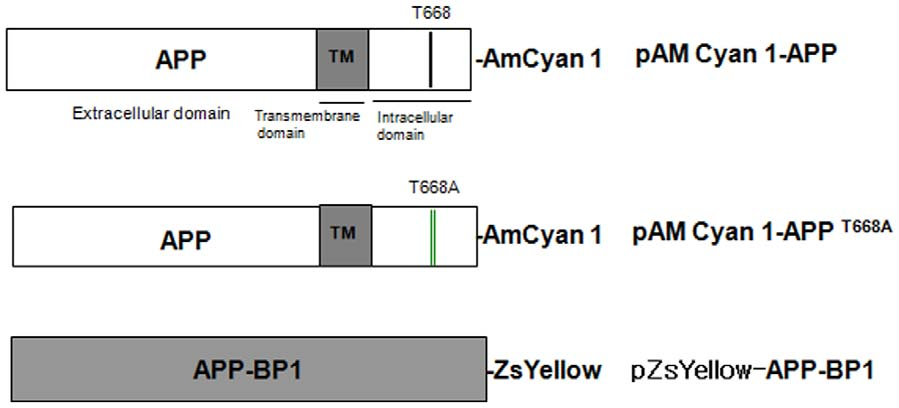
A list of DNA constructs used in FRET experiments. A list of DNA constructs used in FRET study (Figures 8–10) was shown.

**Figure 8:**
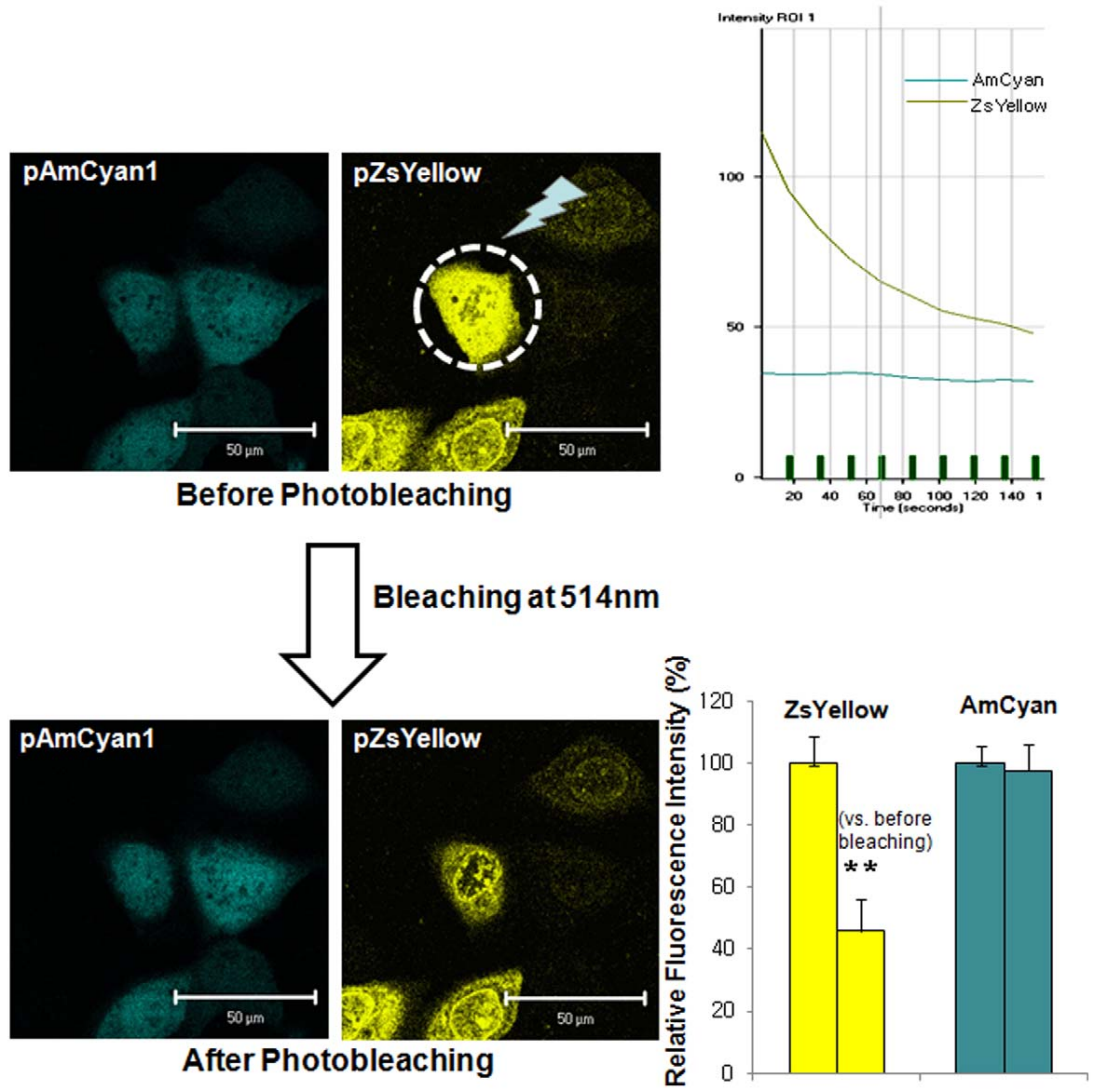
Molecular interaction between pZsYellow vector and pAMCyan1 vector was examined by FRET. HEK 293 cells on coverlips in 6-well plates were transiently co-transfected with pZsYellow vector and pAMCyan1 vector. Figures in the left panel show representative results from the seven independent experiments. The right upper panel shows a representative graph indicating the time-lapse fluorescence changes in the region of interest (ROI). Green line: AMCyan1 fluorescence; gray: ZsYellow throughout the progression of successive bleaching marked as vertical green bars. In the right lower panel, relative fluorescence intensity is denoted after standardization of intensities before and after photobleaching of ZsYellow at 514 nm. *p<0.05 versus fluorescence intensity before photobleaching of ZsYellow at 514 nm (by Student's t-test). Scale bars = 50 µm.

**Figure 9 pone-0014203-g009:**
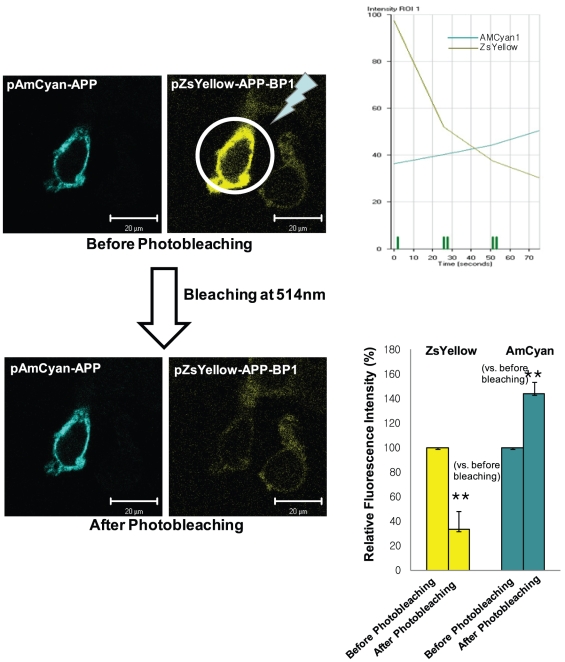
Molecular interaction between APP-BP1 and APP was examined by FRET. HEK 293 cells on coverlips in 6-well plates were transiently co-transfected with pZsYellow-APP-BP1 and pAMCyan1-APP. Figures in the left panel show representative results from the seven independent experiments. The right upper panel shows a representative graph indicating the time-lapse fluorescence changes in the region of interest (ROI). Green line: AMCyan1 fluorescence; gray: ZsYellow throughout the progression of successive bleaching marked as vertical green bars. In the right lower panel, relative fluorescence intensity is denoted after standardization of intensities before and after photobleaching of ZsYellow at 514 nm. *p<0.05 versus fluorescence intensity before photobleaching of ZsYellow at 514 nm (by Student's t-test). Scale bars = 50 µm.

**Figure 10:**
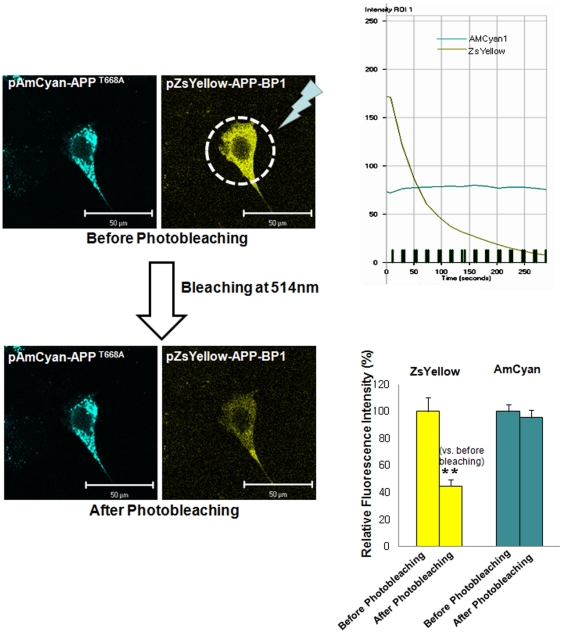
Molecular interaction between APP-BP1 and APP^T668A^ was examined by FRET. HEK 293 cells on coverlips in 6-well plates were transiently co-transfected with pZsYellow-APP-BP1 and pAMCyan1-APP^T668A^. Figures in the left panel show representative results from the seven independent experiments. The right upper panel shows a representative graph indicating the time-lapse fluorescence changes in the region of interest (ROI). Green line: AMCyan1 fluorescence; gray: ZsYellow throughout the progression of successive bleaching marked as vertical green bars. In the right lower panel, relative fluorescence intensity is denoted after standardization of intensities before and after photobleaching of ZsYellow at 514 nm. *p<0.05 versus fluorescence intensity before photobleaching of ZsYellow at 514 nm (by Student's t-test). Scale bars = 50 µm.

APP-BP1 has been reported to interact with the intracellular C-terminal domain of APP [4], however, the physiological significance of this interaction remains unclear. According to the cell cycle progression, APP is thought to be phosphorylated at threonine 668. During the G2/M phase, APP phosphorylation is maximally induced by cdc2 kinase at threonine 668 [24]. We also confirmed that APP is phosphorylated at threonine 668 during the G2/M phase in SHSY5Y cells (Figure S2 and S3). We hypothesized that the interaction between APP and APP-BP1 might be regulated by APP phosphorylation at threonine 668. To test this theory, we examined the effect of threonine 668 phosphorylation on the APP/APP-BP1 interaction using a FRET method to compare wild-type and mutant clones: APP-BP1 cloned in a pZsYellow- N1 vector, APP in a pAMCyan1- N1 vector, and a point mutation APP^T668A^ in a p pAMCyan1- N1 vector (Fig. 7). HEK293 cells on coverslips in 6-well plates were transiently co-transfected with one of the following combinations: 1) pAMCyan1-N1 vector and pZsYellow-N1 vector, 2) pAMCyan 1- APP and pZsYellow- APP-BP1, 3) pAMCyan1-APP ^T668A^ and pZsYellow-APP-BP1 (Figs. 8, 9, 10). At 24 h after transfection, the coverslips were mounted on an Axiovert 200 inverted microscope (Zeiss, Jena Germany). The energy transfer was detected as an increase in donor fluorescence (AMCyan1) after complete photobleaching of the acceptor molecules (ZsYellow). Cells showing both AMCyan1 and ZsYellow signals were photobleached at 514 nm (laser power 100%) to destroy the acceptor molecules. The cells were then rescanned using 458-nm light and an increase of the AMCyan1 signal within the photobleached area was used as a measure of FRET. As shown in Fig. 8, the cells co-expressing pAMCyan1 and pZsYellow vectors only showed no change in AMCyan1 donor signal after photobleaching of the ZsYellow signal at 514 nm. In Fig. 9, AMCyan1 donor signal was significantly increased after complete photobleaching of ZsYellow signal at 514 nm, indicating that FRET is present between APP-BP1 and wild -type APP. By contrast, FRET was not observed between pZsYellow-APP-BP1 and pAMCyan1-APP^T668A^ (Fig. 10). It was confirmed that the subcellular localization of APP and APP^T668A^ showed no difference, which excluded the possibility that FRET would be affected by altered subcellular localization due to the mutation of APP at threonine 668 (Figure S4). These results suggest that the interaction between the two proteins is dependent upon the APP phosphorylation at threonine 668, and that the site-specific phopshorylation inhibits the interaction between the two proteins.

## Discussion

Since APP-BP1 was first identified as a protein that interacts with APP [4], numerous studies have investigated its functions as well as its possible pathological roles in AD [22]. APP-BP1 has been reported to be one component of bipartite enzyme complex, together with hUba3 and to be involved in SCF complex activation [8],[9],[11]. Exogenous expression of APP-BP1 in ts41 cells containing the phenotype which leads to successive S phases of the cell cycle without intervening G(2), M, and G(1), was shown to drive the cell cycle through the S-M checkpoint and this function requires both hUba3 and hUbc12 [25]. Overexpression of APP-BP1 causes DNA synthesis and apoptosis in primary neurons [22],[25]. In the mean time, APP-BP1 downregulates Aβ_1–42_ production by interacting with PS1 under physiological conditions in primary neurons [26].

In this study, we investigated the normal physiological role for APP-BP1 in the cell cycle progression of fetal neural stem cells. APP-BP1 knockdown by siRNA treatment was found to inhibit the cell cycle transition into S phase, resulting in G1 phase arrest (as shown in Fig. 1). We also confirmed that BrdU-incorporated cells were significantly decreased in APP-BP1 siRNA-transfected fetal neural stem cells (Fig. 2), indicating that proliferation was reduced in APP-BP1 siRNA-treated fetal neural stem cells. Interestingly, APP knockdown was found to increase the proliferation of fetal neural stem cells (Fig. 3). These results strongly suggest that APP-BP1 modulates the cell cycle progression of fetal neural stem cells and that APP antagonistically regulates the function of APP-BP1 in cell cycle progression from G1 to S Phase.

We next investigated the changes in APP-BP1 protein levels during different cell cycle phases, and we found that APP-BP1 levels, measured through Western blotting, were highly increased in the S phase of fetal neural stem cells (Fig. 5). These results indicate that APP-BP1 is required for the transition from G1 to S phase, and that its expression is increased at S phase in fetal neural stem cells.

Previously, dAPP-BP1 (a *Drosophila* homologue of APP-BP1) has been reported to interact antagonistically with APP-like protein (APPL) during *Drosophila* development. These reports showed that a null mutation of dAPP-BP1 blocks the NEDD8 conjugation pathway and causes apoptosis in imaginal disc cells. Furthermore, APPL overexpression inhibits the NEDD 8 conjugation pathway, disrupts the normal bristle pattern in the fly thorax, and induces apoptosis in wing imaginal discs [26].

In the present study, we used an ISH method to examine the expression patterns of both APP-BP1 and APP during rat developmental stages. While immunohistochemical analyses of APP expression during various developmental stages in rats have been reported [27], ours is the first report of the expression profile of APP-BP1 during developmental stages in mammals.

We found a moderate, ubiquitous level of APP-BP1 mRNA signal in developing embryonic and early postnatal brains (Fig. 6A–D), but APP-BP1 expression appeared to be reduced by P12 (Fig. 6D), and only a marginal signal of APP-BP1 was found in the adult brain (Fig. 6E). We were especially interested in whether APP-BP1 and APP colocalize in VZ, where neural precursor cells proliferate. In the cerebral cortex of E16 rat, substantial signals for both APP-BP1 (Fig. 6K) and APP (Fig. 6L) mRNAs were observed in VZ.

It has been reported that the last 31 C-terminal amino acids of APP are critical for the interaction with APP-BP1 [22]. APP contains several putative phosphorylation sites within its cytoplasmic domain. Among them, threonine 668 was reported to be phosphorylated in a cell cycle- dependent manner during the G2/M phase [24]. Here, we tested whether the interaction between APP-BP1 and APP was affected by phosphorylation at threonine 668 by FRET assay. We found that the interaction of APP-BP1 and APP is mediated by phosphorylation of APP at threonine 668. When APP-BP1 in the pZsYellow vector and APP^T668A^ in the pAMCyan1 vector were co-transfected, no FRET was observed (Fig. 10), and when APP-BP1 in the pZsYellow vector was co-transfected with wild-type APP in the pAMCyan1 vector, AMCyan1 fluorescence was significantly increased after photobleaching at 514 nm (Fig. 9). These results show that the phosphorylation of APP at threonine 668 is required for the interaction with APP-BP1.

In the present study, we found that APP-BP1 plays a role in the cell cycle progression of fetal neural stem cells. Furthermore this effect appears to be antagonistically regulated by APP. In addition, we found a moderate ubiquitous level of APP-BP1 mRNA in developing embryonic and early postnatal brains; however, APP-BP1 expression is reduced by P12, and only low levels of APP-BP1 were found in the adult brain. In the cerebral cortex of E16 rats, substantial expression of both APP-BP1 and APP mRNAs was observed in the ventricular zone.

## Materials and Methods

### Reagents and antibodies

Anti-APP-BP1 and anti-GAPDH (FL-335) antibodies were obtained from Santa Cruz Biotechnology (CA, USA); Anti-APP (6E10) antibody, from Chemicon (CA, USA). The source of siRNA for APP-BP1 (SMARTpool® reagent) was Dharmacon (CO, USA). pAM Cyan1-N1 and pZsYellow- N1 vectors were obtained from Clontech (CA, USA).

### Fetal neural stem cell culture

Fetal neural stem cell culture was performed as previously described [28]. C57BL6 mouse fetal cortex was dissected at gestation days 13, TP13 (timed pregnant 13; Japan SLC. Inc, Haruno Breeding branch). Cells were isolated by mechanical dissociation in MEM (minimum essential medium) supplemented with 1M HEPES, 1% penicillin/streptomycin (pH 7.2), and 10^7^ cells were plated in T75 flask and the generation of neural sphere derived from fetal neural stem cells was performed as previously described [29]. Briefly, fetal neural stem cells were aggregated to a neurosphere, whereupon, cells were allowed to proliferate in the proliferating media, Dulbecco's modified Eagle's medium/F-12 (1 ∶ 1) (Gibco, Grand Island, NY, USA) medium supplemented with 2 mmol/L l-glutamine (Gibco), 0.6% glucose, 5 µmol/L HEPES, 25 µg/mL insulin, 100 µg/mL apo-transferrin, 30 nmol/L sodium selenite, 100 nmol/L putresine, and 20 nmol/L progesterone (all supplements purchased from Sigma, MO, USA) with 10 ng/mL recombinant basic fibroblast growth factor (bFGF; Roche, Mannheim, Germany) and 20 ng/mL epidermal growth factor (EGF; BD sciences, MA, USA) for 4 days. For BrdU incorporation assessment, we dissociated the neurospheres and subcultured fetal neural stem cells (5×10^5^ cells/well) in 6-well plates, in proliferating media, and then treated the cells with 10 nM siRNAs. After 24 h, the cells were treated with BrdU (1 µM) for 24 h. Then we transferred the cells to new 6-well plates that contained 12 mm glass coverslips (Marienfeld, Lauda-Konlgshofen, Germany) pre-coated with laminin (Roche, Mannheim, Germany) at the concentration of 20 µg/ml. The cells were cultured in the differentiation media without growth factors (EGF and bFGF) for 30 min and assessed with immunocytochemistry using an anti BrdU-antibody.

### Immunocytochemistry for BrdU staining

Cells were fixed in 4% paraformaldehyde for 20 min at room temperature. They were washed in PBS containing 0.3% Triton X-100 for 5 min, 3 times for permeabilization and treated 1N HCl for 10 min on ice and then 2N HCl for 10 min on room temperature for DNA denaturation. After three times washing in PBS for 5 min, cells were treated with 0.1M borate buffer (pH 8.5) for 12 min at room temperature. After three washes in perrmeabilization buffer, they were blocked by 5% normal goat serum in permiabilization buffer for 1 h at room temperature. And then cells were incubated with BrdU primary antibody (final concentration: 5 µg/ml) diluted in permeabilization buffer for overnight at 4°C. After three washes, primary antibodies were revealed by incubating the cells for 1 h with Cy3 conjugated secondary antibody at a dilution ratio of 1∶300 (Jackson ImmunoResearch, PA, USA). After three washes in PBS, cells were mounted on microscope slides in mounting medium (DAKO, CA, USA) that included DAPI (1 µM) for nuclear staining. Cells were then observed under a Confocal microscope (LSM 510, Zeiss, Germany). For BrdU staining experiment, totally fifteen pregnant mice were used for culturing fetal neural stem cells. Five independent experiments were performed for each experiment.

### DNA constructs and mutagenesis

Human APP-BP1 cDNA included in the pcDNA3 vector was kindly provided by Dr. Rachael L. Neve at Harvard Medical School. The constructs of pAM Cyan 1-APP and pZsYellow-APP-BP1 were generated by PCR from human APP-BP1 in the pcDNA3 vector and human APP in the pCB6 vector, respectively. To construct the T668A post mutation of APP, we used a QuickChange Site-Directed Mutagenesis Kit (Stratagene, CA, USA).

### Cell cycle analysis

Fetal neural stem cells were synchronized to G1 phase by treatment with 2 mM thymidine for 16 h, and then released from cell cycle arrest by replacing the non thymidine-containing media. Cells were harvested at 0, 2, 4, 6, 8, 10 and 12 h, and APP-BP1 protein levels and DNA contents were examined by Western blotting and flow cytometry, respectively. Harvested cells were washed three times with phosphate-buffered saline (PBS), fixed in 70% ethanol, stained with propidium iodide (PI, 25 µg/mL) (Sigma), and incubated for 30 min at 37°C with RNase A (20 µg/mL) (Roche Applied Science, USA). Cellular DNA content was then evaluated by flow cytometry with a fluorescence activated sorting (FACS) Scan instrument (BD Biosciences).

### Western blotting

Proteins were resolved in sodium dodecyl sulfate (SDS) polyacrylamide gels, electrophoresed with 30–50 µg protein/lane, and transferred onto a nitrocellulose membrane (GE Healthcare AB, Sweden). The protein blot was confirmed with anti-APP-BP1 and anti-GAPDH antibodies at final concentrations of 0.2 and 0.2 µg/ml, respectively, and detected with a horseradish peroxidase-conjugated secondary antibody (GE Healthcare AB, Sweden) at a dilution ratio of 1∶2000. Immunoreactive bands were visualized with an enhanced chemiluminescence system (ECL; GE Healthcare AB, Sweden).

### Fluorescence resonance energy transfer (FRET)

FRET measurements were observed using a Zeiss LSM 510 confocal microscope mounted on Zeiss Axiovert 200 inverted microscope. FRET was measured by employing a method developed for laser scanning confocal microscopy that usesa an argon laser to excite Cyan1 or ZsYellow. HEK293 cells on 6 well plates were co-transfected with one of the following combinations: 1) pAMCyan1-N1 vector and pZsYellow-N1 vector, 2) pAMCyan 1- APP and pZsYellow- APP-BP1, 3) pAMCyan1-APP^T668A^ and pZsYellow-APP-BP1 using Fugene 6 (Roche Molecular Biochemicals, Germany). 24 h after transfection, the coverslips were mounted on the Axiovert 200 inverted microscope (Zeiss, Jena Germany). An initial scan was obtained at low energy using the 458 nm line of an argon laser to record the AMCyan1 signal. A second scan was performed with the 568 nm line, and the co-localization of cells was recorded. Cells were then photobleached with intense 514 nm light (laser power 100%) to destroy the acceptor molecules. Energy transfer was detected as an increase in donor fluorescence (AMCyan1) after complete photobleaching of the acceptor molecules (ZsYellow) at 514 nm. The amount of energy transfer was calculated as the percent increase in donor fluorescence after acceptor photobleaching. An increase of the ZsYellow signal within the photobleached area was used as a measure of the amount of FRET present.

### In situ hybridization

*In situ* hybridization (ISH) was carried out as previously described [30]. Embryos and postnatal brains from rats were prepared at different developmental stages. Frozen sections (12 µm) were cut sagitally (embryo) or horizontally (postnatal brains), post-fixed with 4% paraformaldehyde, treated with 0.25% acetic anhydride in 0.1 M triethanolamine/0.9% NaCl, and dehydrated in ethanol/chloroform. The probes used in this study were generated against the 234–723 nucleotide sequences of APP (GenBank No, NM 019288), and the 299–815 nucleotide sequences of APP-BP1 (GenBank No, NM032072) in the presence of α-[35S] UTP (1000–1500 Ci/mmol, GE Healthcare AB, Sweden). Sections were hybridized overnight at 53°C with the labeled probe. The following day, the sections were treated with RNase A (20 mg/mL, Roche Germany) for 30 min at 25°C, and washed in a series of SSC baths: 60 min in 2× SSC at 50°C, 60 min in 0.2× SSC at 55°C, and 60 min in 0.2× SSC at 60°C. The sections were then rinsed briefly in a graded series of ethanols baths containing 0.3 M ammonium acetate. Finally, sections were then exposed to β-max film (GE Healthcare AB, Sweden) for 6 days. To examine cell-level distribution of ISH signals, some slides were dipped into Kodak NTB2 photoemulsions, exposed, developed, lightly counterstained with cresyl violet, and cover-slipped. Experiments were duplicated.

### Statistical Analysis

Data are expressed as mean ± SEM values. One-way ANOVA and Student's *t*-tests (SPSS, Chicago, IL) were used for determining statistical significance. Results were considered statistically significantly for *p*<0.05.

## Supporting Information

Figure S1 The effect of siRNA for APP was detected by Western blotting. (A) After the treatment of fetal neural stem cells with 10 nM of non-targeting and APP-BP1 siRNAs for 72 h, APP-BP1 protein level was examined by Western blotting. Densitometrical analysis was also performed (* p<0.05). (B) After treatment of fetal neural stem cells with 10 nM of non-targeting and APP siRNAs for 72 h, APP protein level was examined by Western blotting.
(1.44 MB TIF)

Figure S2 Phosphorylation of APP at threonine 668 was examined according to cell cycle phases SH-SY5Y cells were synchronized to the G1 phase by treatment with 2 mM thymidine for 16 h, and then released from cell cycle arrest by replacing the thymidine-containing media. Cells were harvested at 0, 2, 4, 6, 8, 10 and 12 h and the protein level of APP phosphorylated at threonine 668 was examined by Western blotting.
(0.10 MB TIF)

Figure S3 Cell cycle analysis of SH-SY5Y cells following cell cycle synchronization by thymidine treatment SH-SY5Y cells were synchronized to the G1 phase by treatment with 2 mM thymidine for 16 h, and then released from cell cycle arrest by replacing the thymidine-containing media. SH-SY5Y cells were synchronized to the G1 phase by treatment with 2 mM thymidine for 16 h, and then released from cell cycle arrest by replacing the thymidine-containing media. Cells were harvested at 0, 2, 4, 6, 8, 10 and 12 h and cell cycle was analysed by FACS.
(0.16 MB TIF)

Figure S4 Confirmation of subcellular localization of APP and APPT668A. HEK 293 cells on coverlips in 6-well plates were transiently transfected with pAMCyan1-APP or APPT668A. 24 h after transfection, the subcellular localization was examined using Zeiss LSM 510 confocal microscope. Scale bars = 50 µm. Representative images were shown.
(0.25 MB TIF)
