# Supplementary figures and images for: Amyloid Precursor Protein Binding Protein-1 Modulates Cell Cycle Progression in Fetal Neural Stem Cells
Source: PLoS One. 2010 Dec 2;5(12):e14203. doi: 10.1371/journal.pone.0014203 (PMC2996309; doi:10.1371/journal.pone.0014203)

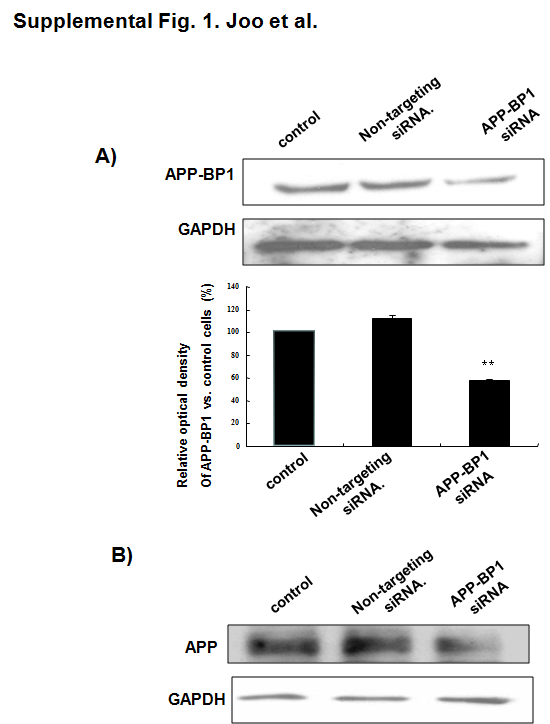

Supplement: Figure S1 — The effect of siRNA for APP was detected by Western blotting. (A) After the treatment of fetal neural stem cells with 10 nM of non-targeting and APP-BP1 siRNAs for 72 h, APP-BP1 protein level was examined by Western blotting. Densitometrical analysis was also performed (* p<0.05). (B) After treatment of fetal neural stem cells with 10 nM of non-targeting and APP siRNAs for 72 h, APP protein level was examined by Western blotting. (1.44 MB TIF) [file pone.0014203.s001.tif]

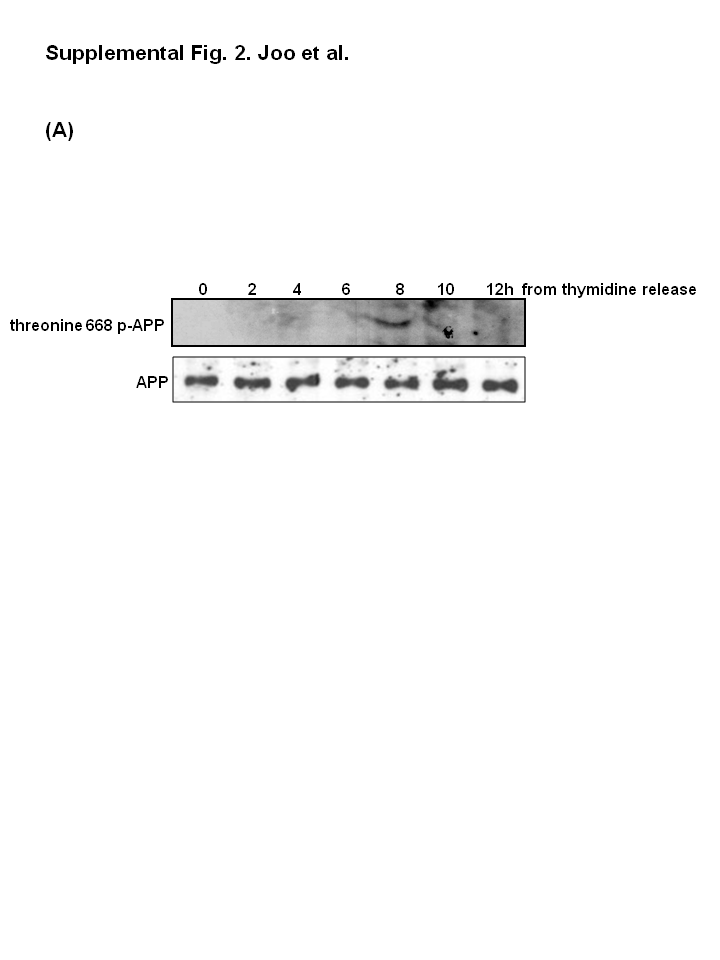

Supplement: Figure S2 — Phosphorylation of APP at threonine 668 was examined according to cell cycle phases SH-SY5Y cells were synchronized to the G1 phase by treatment with 2 mM thymidine for 16 h, and then released from cell cycle arrest by replacing the thymidine-containing media. Cells were harvested at 0, 2, 4, 6, 8, 10 and 12 h and the protein level of APP phosphorylated at threonine 668 was examined by Western blotting. (0.10 MB TIF) [file pone.0014203.s002.tif]

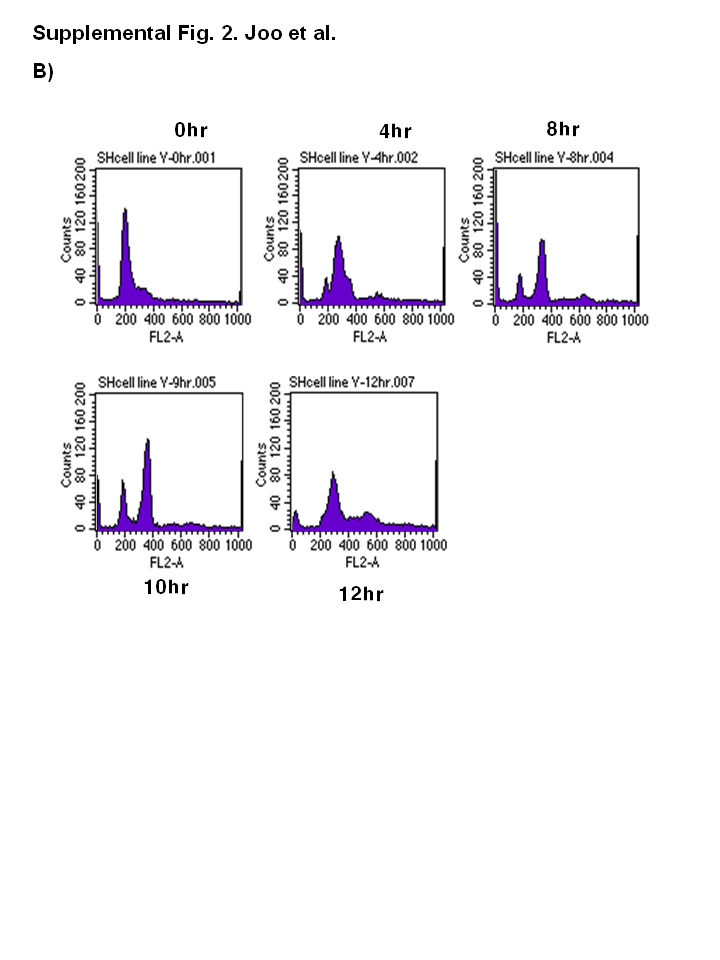

Supplement: Figure S3 — Cell cycle analysis of SH-SY5Y cells following cell cycle synchronization by thymidine treatment SH-SY5Y cells were synchronized to the G1 phase by treatment with 2 mM thymidine for 16 h, and then released from cell cycle arrest by replacing the thymidine-containing media. SH-SY5Y cells were synchronized to the G1 phase by treatment with 2 mM thymidine for 16 h, and then released from cell cycle arrest by replacing the thymidine-containing media. Cells were harvested at 0, 2, 4, 6, 8, 10 and 12 h and cell cycle was analysed by FACS. (0.16 MB TIF) [file pone.0014203.s003.tif]

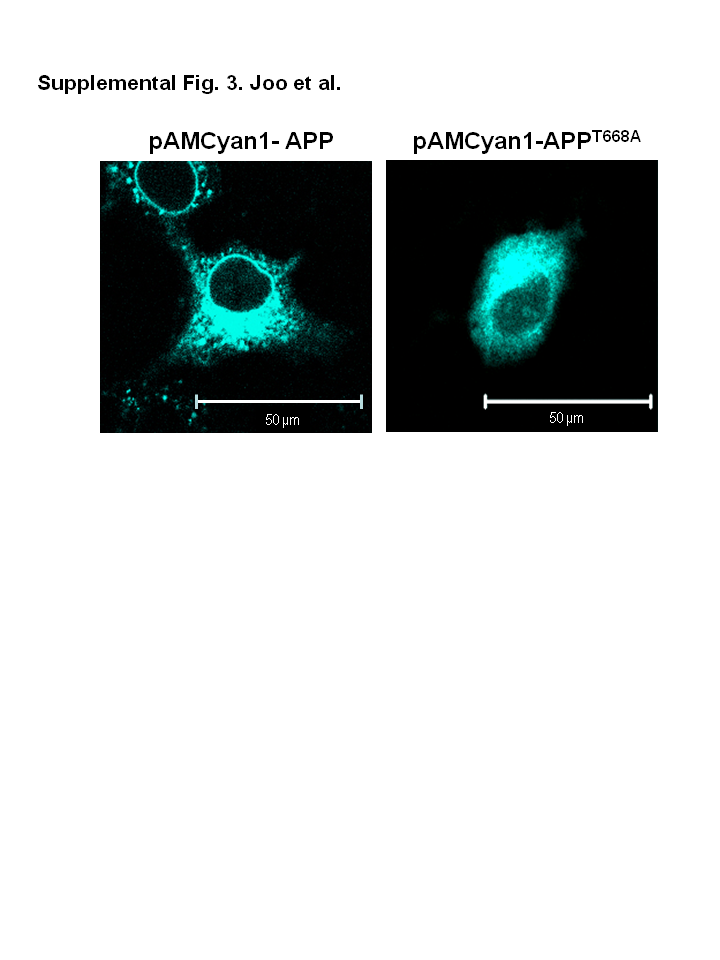

Supplement: Figure S4 — Confirmation of subcellular localization of APP and APPT668A. HEK 293 cells on coverlips in 6-well plates were transiently transfected with pAMCyan1-APP or APPT668A. 24 h after transfection, the subcellular localization was examined using Zeiss LSM 510 confocal microscope. Scale bars = 50 µm. Representative images were shown. (0.25 MB TIF) [file pone.0014203.s004.tif]
